# Predictive Modeling and Analyses of National Emergency Department Data for Improving Patient Outcomes of Nephrolithiasis

**DOI:** 10.7759/cureus.40297

**Published:** 2023-06-12

**Authors:** Kori L Stewart

**Affiliations:** 1 Diagnostic Imaging, Health Informatics, Quinnipiac University, Hamden, USA

**Keywords:** urology emergency, predictive modeling, plasma osmolality, dehydration, irritable bowel syndrome, ultrasound, computed tomography, medical imaging, kidney stones, nephrolithiasis

## Abstract

Background: Nephrolithiasis, or kidney stones, imposes a significant burden of disease in the United States and comes with considerable costs, pain, and morbidity. The exact cause of stone formation is undefined, but the formation is a process. Risk factors include environmental, diabetes, obesity, metabolic syndromes, low fluid intake, dehydration, diet, inflammatory bowel disorders, irritable bowel syndrome (IBS), and genetics. Laboratory testing and appropriate diagnostic imaging studies are two key components of assessment and prevention.

Methods: This is a retrospective, quantitative study utilizing the Healthcare Cost and Utilization Project’s (HCUP) National Emergency Department Sample (NEDS)'s existing databases from 2012 to 2014 to classify outcomes for nephrolithiasis patients. International Classification of Diseases, Ninth Revision (ICD-9-CM) billing codes related to nephrolithiasis, relevant medical imaging exams, and procedural and surgical billing codes for interventions and procedures were selected. Descriptive statistical analyses as well as multiple regression models were used to analyze frequencies and percentages of variables and the relationship of the data, identify co-linearity among variables, and predict outcomes.

Results: The study sample includes a total of 509,192 emergency department (ED) visits for nephrolithiasis from 2012 to 2014 and reveals that IBS patients are two times more likely to require intervention. Stepwise regression models yield P-values of 0.004 for gender, 0.017 and 0.018 for minor diagnostic procedures, 0.006 and 0.001 for minor therapeutic procedures, and 0.000 and 0.001 for major therapeutic procedures when predicting total cost of care, and have a statistically significant impact on patient outcomes of nephrolithiasis.

Conclusions: This research offers an investigation of the prevalence of nephrolithiasis based on age, gender, and co-morbidity, specifically IBS, and is the first to report on patient outcomes. This analysis also provides clinicians with recommendations to utilize for a comprehensive assessment of nephrolithiasis patients in the ED.

## Introduction

Nephrolithiasis, or kidney stones, imposes a significant burden of disease in the United States. Nearly one in every 11 patients of working age will experience nephrolithiasis and 50% will encounter recurrence [1]. The formation of nephrolithiasis is a process, and not a single event, in which crystallization and crystal aggregation of minerals from supersaturated urine occur. Nephrolithiasis can be composed of calcium oxalate, cystine, uric acid, or struvite. The most common type of stone formed is composed of calcium oxalate. Urine concentration, infection, and urinary pH are inherent factors in this crystallization process and nephrolithiasis formation [2].

The National Institute of Health Consensus Development Conference on the Prevention and Treatment of Kidney Stones outlines that all stone formers, whether single stone or recurrent, should experience at a minimum a 24-hour urine collection [3]. Laboratory evaluation for nephrolithiasis also may include blood urea nitrogen, creatinine, serum electrolytes, calcium, phosphorus, and uric acid, in addition to parathyroid hormone levels, vitamin D, serum bicarbonate concentration, and urine pH levels, all of which are strong indications for stone risk.

Medical imaging modalities utilized for the diagnosis of nephrolithiasis include computed tomography (CT), ultrasound, and magnetic resonance imaging (MRI), with intravenous pyelogram (IVP) having been used historically. Diagnostic imaging is essential in the clinical decision-making process for nephrolithiasis treatment and prevention of recurrence. CT is the recommended modality for initial imaging of suspected nephrolithiasis due to its high sensitivity and specificity by the American College of Radiology (ACR); however, ultrasound is often the first modality utilized at the point of care. Low-dose CT scans are recommended for patients with a body mass index (BMI) of 30 kg/m^2^ and a full-dose scan is recommended for those with a greater BMI [4]. Although ultrasound is an acceptable means of imaging nephrolithiasis and a primary means of imaging pediatric and pregnant patients in the first trimester, this modality has its limitations. Often ultrasound over- or underestimates stone size, presenting physicians with incorrect information and potentially delaying proper treatment of the patient. 

Upon presentation to the ED, patients with suspected first-time nephrolithiasis or recurrent stones should be evaluated with a complete laboratory and diagnostic imaging workup. Chronic dehydration is easily detected with plasma osmolality and copeptin levels, which can easily be determined with laboratory testing. Certain comorbidities associated with nephrolithiasis produce larger stones, specifically in patients with inflammatory bowel disease/disorders (i.e. IBD and irritable bowel syndrome [IBS]). IBD and IBS patients have a greater risk of nephrolithiasis due to fluid imbalance and dehydration due to chronic and acute gastrointestinal symptoms. Hydration status (detected with plasma osmolality) and comorbidities play an integral role in stone size and these factors should be used to guide the physician in the care of the nephrolithiasis patient, thus improving patient outcomes and decreasing overall healthcare costs.

## Materials and methods

This is a retrospective, quantitative study utilizing the Healthcare Cost and Utilization Project’s (HCUP) National Emergency Department Sample (NEDS) databases from 2012 to 2014 to classify outcomes for nephrolithiasis patients. This study analyzed frequencies and percentages of variables, as well as the relationship of data selected with multiple regression models and identification of co-linearity among the variables using IBM SPSS Statistics version 26 (IBM Corp, Armonk, NY). The key objective was to be able to analyze and produce a predictive model for improving patient outcomes of nephrolithiasis.

ED visits in patients 18 years of age and older that have International Classification of Diseases, Ninth Revision, ICD-9-CM, billing codes related to nephrolithiasis, calculus of kidney (592.0), calculus of ureter (592.1), calculus unspecified (592.9), calculus in diverticulum of the bladder (594.0), other calculus in the bladder (594.1), calculus in the urethra (594.2), other lower urinary tract calculus (594.8), calculus of lower urinary tract unspecified (594.9), uric acid nephrolithiasis (274.11), as well as hematuria (599.71), hydronephrosis (591.0), renal colic (788.0), dehydration (276.51), and inflammatory bowel disorders (564.1 irritable bowel syndrome, 555 enteritis, and 556 ulcerative colitis) were identified. Relevant medical imaging exams billed including computed axial tomography (CT) exams of the abdomen (88.01, 88.02) and kidney (87.71), general radiography of the abdomen (88.19), abnormal findings on radiography exam (793), IVP (87.73), as well as an ultrasound of the abdomen (88.76) and ultrasound of the urinary system (88.75) were utilized in this study. Finally, procedural and surgical billing codes for ureteroscopy (56.31), cystoscopy (57.32), ultrasonic fragmentation of urinary stones (59.95), retrograde pyelogram (87.74), percutaneous pyelogram (87.75), extracorporeal shockwave lithotripsy (ESWL) (98.5), and ESWL of kidney, ureter and/or bladder (98.51) were utilized.

Multiple patient-level variables from the HCUP core datasets including age, gender, and comorbidities of obesity, diabetes, IBS, dehydration, and hyperosmolality, as well as relevant medical imaging exams and procedures in addition to total charges and discharge versus admission were extracted. 

Statistical analysis was performed utilizing IBM SPSS Statistics version 26 on an OS X Yosemite operating system. A statistical analysis of the NEDS core data sets from 2012 to 2014 was done using descriptive analytics of frequencies and percentages which provides a detailed summary of the data sets. Regression analyses were performed in SPSS to determine the variables for use and to ensure the model fits the data. A linear, or standard, regression method was utilized, as well as stepwise regression models.

## Results

HCUP NEDS datasets for 2012, 2013, and 2014 were analyzed for patients with a primary, secondary, tertiary, or quaternary diagnosis of nephrolithiasis. The results measure frequencies and percentages of variables with descriptive statistical analysis which provide insight into whether there are statistically significant numerical observations within the datasets. The total number of nephrolithiasis ED visits investigated in this study is 509,192.

Descriptive statistical analysis

Analyses of the HCUP NEDS databases have identified 164,410 ED visits for nephrolithiasis in 2012, 163,254 in 2013, and 181,528 in 2014 (see Table 1).

**Table 1:** Primary Diagnoses in Nephrolithiasis Cases

2012					
		Diagnosis 1	Diagnosis 2	Diagnosis 3	Diagnosis 4
N	Valid	164410	164410	164410	164410
	Missing	0	0	0	0
Diagnosis 1					
		Frequency	Percent	Valid Percent	Cumulative Percent
	5641	38	0.0	0.0	4.8
	591	447	0.3	0.3	8.1
	5920	103037	62.7	62.7	70.7
	5921	12894	7.8	7.8	78.6
	7880	3139	1.9	1.9	89.6
	V1301	3	0.0	0.0	99.9
2013					
		Diagnosis 1	Diagnosis 2	Diagnosis 3	Diagnosis 4
N	Valid	163254	163254	163254	163254
	Missing	0	0	0	0
Diagnosis 1					
		Frequency	Percent	Valid Percent	Cumulative Percent
	5641	30	0.0	0.0	5.0
	591	548	0.3	0.3	8.2
	5920	103612	63.5	63.5	71.6
	5921	12832	7.9	7.9	79.5
	7880	2903	1.8	1.8	90.3
	V1301	8	0.0	0.0	99.9
2014					
		Diagnosis 1	Diagnosis 2	Diagnosis 3	Diagnosis 4
N	Valid	181528	181528	181528	181528
	Missing	0	0	0	0
Diagnosis 1					
		Frequency	Percent	Valid Percent	Cumulative Percent
	5641	37	0.0	0.0	5.5
	591	748	0.4	0.4	8.9
	5920	115624	63.7	63.7	72.6
	5921	13472	7.4	7.4	80.0
	7880	2946	1.6	1.6	90.8
	V1301	7	0.0	0.0	99.9

Nephrolithiasis is identified as the principal diagnosis, or chief responsible condition for hospital care, in approximately 71% of these visits across 2012, 2013, and 2014. The characteristics of nephrolithiasis patients identified exhibit an average age for peak of incidence between 30 and 42 years over the three-year period with slightly greater frequency among males (53.8% in 2012; 53.6% in 2013; 53.1% in 2014) than females (46.2% in 2012; 46.4% in 2013; 46.9% in 2014). Secondary diagnoses aside from nephrolithiasis in this population include hydronephrosis, IBS, and a history of renal calculi. Results from descriptive analyses used in this study provide additional insight into the HCUP NEDS database. Initial cases selected were filtered for IBD and IBS. Interestingly, there were minimal cases of nephrolithiasis patients returned with IBD (ulcerative colitis, enteritis) as compared to IBS, and, therefore, the principal focus of this research is on IBS patients.

The prevalence of nephrolithiasis in patients with IBS across the three years analyzed presented 359 patients in 2012, 272 in 2013, and 327 patients in 2014 for a total of 958 patients. Patients with a documented comorbidity make up 0.19% of this nephrolithiasis population, with a lower incidence observed in males (28.1% in 2012; 32% in 2013; 32.7% in 2014) than that of females (71.9% in 2012; 68% in 2013; 67.3% in 2014) (see Table 2). Therefore, gender in IBS patients should be considered a key component of nephrolithiasis. 

**Table 2:** Prevalence of Irritable Bowel Syndrome in Males vs. Females

2012					
Indicator of Sex					
		Frequency	Percent	Valid Percent	Cumulative Percent
Valid	0	101	28.1	28.1	28.1
	1	258	71.9	71.9	100.0
	Total	359	100.0	100.0	
2013					
Indicator of Sex					
		Frequency	Percent	Valid Percent	Cumulative Percent
Valid	0	87	32.0	32.0	32.0
	1	185	68.0	68.0	100.0
	Total	272	100.0	100.0	
2014					
Indicator of Sex					
		Frequency	Percent	Valid Percent	Cumulative Percent
Valid	0	107	32.7	32.7	32.7
	1	220	67.3	67.3	100.0
	Total	327	100.0	100.0	

The prevalence of dehydration as a primary diagnosis and/or secondary diagnosis (see Table 3) was investigated as well as hyperosmolality (see Table 4). As the primary recommendation for the prevention of nephrolithiasis is increased fluid intake and based on the process of stone formation, dehydration would likely be present in these patients. Hyperosmolality in patients with nephrolithiasis is worth noting in this study as this is an indicator of dehydration and an alternative method of assessment as plasma osmolality is not identified in ICD-9 coding. As plasma osmolality is a key indicator of chronic dehydration, hyperosmolality is also a key indicator of dehydration. The total number of patients from 2012 to 2014 with a diagnosis of dehydration in this study is 4605 (0.9%). The total number of patients with a diagnosis of hyperosmolality in this study is 204 (0.04%). This low yield results in the question of whether data collection or assessment of hydration status is lacking.

**Table 3:** Prevalence of Dehydration in Nephrolithiasis

2012					
		Diagnosis 1	Diagnosis 2	Diagnosis 3	Diagnosis 4
N	Valid	1624	1624	1624	1624
	Missing	0	0	0	0
Diagnosis 1					
		Frequency	Percent	Valid Percent	Cumulative Percent
	27651	160	9.9	9.9	12.4
	5920	756	46.6	46.6	75.1
	5921	104	6.4	6.4	81.5
Diagnosis 2					
		Frequency	Percent	Valid Percent	Cumulative Percent
	27651	570	35.1	35.1	39.0
	5920	320	19.7	19.7	79.2
	5921	35	2.2	2.2	81.3
2013					
		Diagnosis 1	Diagnosis 2	Diagnosis 3	Diagnosis 4
N	Valid	1402	1402	1402	1402
	Missing	0	0	0	0
Diagnosis 1					
		Frequency	Percent	Valid Percent	Cumulative Percent
	27651	145	10.3	10.3	13.0
	5920	633	45.1	45.1	76.5
	5921	69	4.9	4.9	81.5
Diagnosis 2					
		Frequency	Percent	Valid Percent	Cumulative Percent
	27651	515	36.7	36.7	41.9
	5920	280	20.0	20.0	81.2
	5921	27	1.9	1.9	83.1
2014					
		Diagnosis 1	Diagnosis 2	Diagnosis 3	Diagnosis 4
N	Valid	1579	1579	1579	1579
	Missing	0	0	0	0
Diagnosis 1					
		Frequency	Percent	Valid Percent	Cumulative Percent
	27651	178	11.3	11.3	14.6
	5920	717	45.4	45.4	77.1
	5921	98	6.2	6.2	83.3
Diagnosis 2					
		Frequency	Percent	Valid Percent	Cumulative Percent
	27651	563	35.7	35.7	38.6
	5920	327	20.7	20.7	79.4
	5921	34	2.2	2.2	81.6

**Table 4:** Prevalence of Hyperosmolality in Nephrolithiasis

2012					
		Diagnosis 1	Diagnosis 2	Diagnosis 3	Diagnosis 4
N	Valid	52	52	52	52
	Missing	0	0	0	0
Diagnosis 1					
		Frequency	Percent	Valid Percent	Cumulative Percent
	5920	20	38.5	38.5	88.5
	5921	1	1.9	1.9	90.4
Diagnosis 2					
		Frequency	Percent	Valid Percent	Cumulative Percent
	2760	28	53.8	53.8	57.7
	5920	1	1.9	1.9	88.5
	5921	4	7.7	7.7	96.2
2013					
		Diagnosis 1	Diagnosis 2	Diagnosis 3	Diagnosis 4
N	Valid	70	70	70	70
	Missing	0	0	0	0
Diagnosis 1					
		Frequency	Percent	Valid Percent	Cumulative Percent
	5920	28	40.0	40.0	77.1
	5921	4	5.7	5.7	82.9
Diagnosis 2					
		Frequency	Percent	Valid Percent	Cumulative Percent
	2760	40	57.1	57.1	62.9
2014					
		Diagnosis 1	Diagnosis 2	Diagnosis 3	Diagnosis 4
N	Valid	82	82	82	82
	Missing	0	0	0	0
Diagnosis 1					
		Frequency	Percent	Valid Percent	Cumulative Percent
	5920	33	40.2	40.2	84.1
	5921	2	2.4	2.4	86.6
Diagnosis 2					
		Frequency	Percent	Valid Percent	Cumulative Percent
	2760	48	58.5	58.5	59.8
	5920	3	3.7	3.7	90.2
	5921	1	1.2	1.2	91.5

Medical imaging is a key component of nephrolithiasis assessment and care. Patients included in the HCUP NEDS 2012, 2013, and 2014 Supplemental Emergency Department Files, which provide “information on procedures that were performed in the ED for treat and release” [5], had ultrasound imaging of the urinary system performed as the principal procedure, more than CT imaging, which the ACR’s evidence-based clinical guidelines recommend for initial imaging of suspected nephrolithiasis.

Patients included in the HCUP NEDS Supplemental Inpatient Files, which provide “information on inpatient admissions after ED visits” [5], demonstrate a higher incidence of CT imaging than that of ultrasound demonstrating the need to analyze this data to further support or negate the hypothesis that patients imaged with ultrasound before CT have poorer outcomes and greater overall hospital charges for combined ED and inpatient services. 

The intervention of nephrolithiasis is a strong indicator of stone size as the ability of a smaller stone to pass is far more likely than that of a larger stone which typically results in bleeding or infection requiring intervention. The HCUP NEDS datasets include a procedure class data element that “categorizes ICD-9 procedure codes into one of four broad categories” [6,7]. These categories include minor diagnostic, minor therapeutic, major diagnostic, and major therapeutic. Minor categories represent non-operating room procedures and therapies, whereas major categories represent valid operating room procedures and therapies. Of the nephrolithiasis cases found in the datasets, interventions were performed in 9158 (5.6% of this population) inpatients in 2012, 8542 (5.2% of this population) inpatients in 2013, and 9628 (5.3% of this population) inpatients in 2014 (see Table 5) for a total of 27,328 (5.4% of this overall population) interventions. The 2012 interventions include 2038 minor diagnostic procedures, 3978 minor therapeutic procedures, 58 major diagnostic, and 3048 major therapeutic for those admitted to the hospital with nephrolithiasis (see Table 6). The types of interventions related to nephrolithiasis identified in this study include percutaneous nephrostomy without fragmentation, percutaneous nephrostomy with fragmentation, ureteroscopy, cystoscopy, ultrasonic fragmentation of urinary stones, retrograde pyelogram, percutaneous pyelogram, ESWL, and ESWL of the kidney, ureter, or bladder.

**Table 5:** Principal Procedure Class for Inpatients With Nephrolithiasis

2012					
		Frequency	Percent	Valid Percent	Cumulative Percent
Valid	1	2038	1.2	22.3	22.3
	2	3978	2.4	43.4	65.7
	3	58	0.0	0.6	66.3
	4	3084	1.9	33.7	100.0
	Total	9158	5.6	100.0	
Missing System		155,252	94.4		
Total		164,410	100.0		
2013					
		Frequency	Percent	Valid Percent	Cumulative Percent
Valid	1	1806	1.1	21.1	21.1
	2	3849	2.4	45.1	66.2
	3	43	0.0	0.5	66.7
	4	2844	1.7	33.3	100.0
	Total	8542	5.2	100.0	
Missing System		154,712	94.8		
Total		163,254	100.0		
2014					
		Frequency	Percent	Valid Percent	Cumulative Percent
Valid	1	2024	1.1	21.0	21.0
	2	4391	2.4	45.6	66.6
	3	48	0.0	0.5	67.1
	4	3165	1.7	32.9	100.0
	Total	9628	5.3	100.0	
Missing System		171,900	94.7		
Total		181,528	100.0		

**Table 6:** Principal Interventions and Procedures for Inpatients With Nephrolithiasis

2012				
	Frequency	Percent	Valid Percent	Cumulative Percent
5503	7	1.1	1.1	1.7
5504	4	0.6	0.6	2.3
5631	53	8.0	8.0	80.8
5732	10	1.5	1.5	82.9
5995	5	0.8	0.8	95.3
8774	13	2.0	2.0	97.6
9851	9	1.4	1.4	99.7
2013				
	Frequency	Percent	Valid Percent	Cumulative Percent
5503	238	0.1	0.1	95.9
5504	62	0.0	0.0	96.0
5631	65	0.0	0.0	97.2
5732	171	0.1	0.1	97.3
5995	27	0.0	0.0	98.6
8774	399	0.2	0.2	99.0
8775	21	0.0	0.0	99.0
9851	363	0.2	0.2	99.7
2014				
	Frequency	Percent	Valid Percent	Cumulative Percent
5503	352	0.2	0.2	96.0
5504	92	0.1	0.1	96.0
5631	63	0.0	0.0	97.1
5732	175	0.1	0.1	97.2
5995	51	0.0	0.0	98.5
8774	360	0.2	0.2	98.9
8775	19	0.0	0.0	98.9
9851	373	0.2	0.2	99.6

Total charges for ED services of nephrolithiasis patients versus total charges for inpatients were assessed. The mean for ED services is $5639 in 2012, $5946 in 2013, and $6426 in 2014. The mean for inpatient services is $27,165 in 2012, $29,503 in 2013, and $32,801 in 2014. The significance of cost for patients admitted to the hospital for nephrolithiasis represents additional care, as well as diagnostic and therapeutic procedures.

Descriptive statistical analysis of IBS

Analyses of the 2012, 2013, and 2014 HCUP NEDS databases have identified a total of 958 ED visits for nephrolithiasis in patients with IBS. Although the prevalence of nephrolithiasis was found to be more common in males than females in the initial analyses, the prevalence is far greater in females (67%) with IBS. Of these patients identified, only 22 patients across the three years analyzed were assessed or diagnosed with CT and none were assessed with ultrasound. Of these 958 IBS patients, 104 (10.9% of this population) underwent diagnostic or therapeutic procedures (see Table 7). This number represents a total of 81 minor diagnostic or therapeutic procedures and 27 major diagnostic or therapeutic procedures. Principal interventions and procedures directly related to nephrolithiasis in patients with IBS include a total of 17 in 2012, 16 in 2013, and 11 in 2014 (see Table 8). 

**Table 7:** Principal Procedure Class for IBS Patients IBS, irritable bowel syndrome.

2012					
		Frequency	Percent	Valid Percent	Cumulative Percent
Valid	1	25	7.0	62.5	62.5
	2	10	2.8	25.0	87.5
	4	5	1.4	12.5	100.0
	Total	40	11.1	100.0	
Missing System		319	88.9		
Total		359	100.0		
2013					
		Frequency	Percent	Valid Percent	Cumulative Percent
Valid	1	11	4.0	35.5	35.5
	2	10	3.7	32.3	67.7
	4	10	3.7	32.3	100.0
	Total	31	11.4	100.0	
Missing System		241	88.6		
Total		272	100.0		
2014					
		Frequency	Percent	Valid Percent	Cumulative Percent
Valid	1	15	4.6	45.5	45.5
	2	10	3.1	30.3	75.8
	4	8	2.4	24.2	100.0
	Total	33	10.1	100.0	
Missing System		294	89.9		
Total		327	100.0		

**Table 8:** Principal Interventions and Procedure Total for IBS IBS, irritable bowel syndrome.

2012				
Principal Procedure From Inpatient Discharge Record				
	Frequency	Percent	Percent Valid	Cumulative Percent
5631	1	0.3	0.3	95.3
5732	1	0.3	0.3	95.5
Procedure 2 From Inpatient Discharge Record				
	Frequency	Percent	Percent Valid	Cumulative Percent
5631	1	0.3	0.3	96.1
5732	1	0.3	0.3	96.4
598	4	1.1	1.1	97.5
5995	1	0.3	0.3	97.8
8774	1	0.3	0.3	98.6
Procedure 3 From Inpatient Discharge Record				
	Frequency	Percent	Percent Valid	Cumulative Percent
8774	4	1.1	1.1	99.4
Procedure 4 From Inpatient Discharge Record				
	Frequency	Percent	Percent Valid	Cumulative Percent
5631	1	0.3	0.3	99.2
8774	2	0.6	0.6	99.7
2013				
Principal Procedure From Inpatient Discharge Record				
	Frequency	Percent	Percent Valid	Cumulative Percent
5503	1	0.4	0.4	93.8
9851	1	0.4	0.4	99.3
Procedure 2 From Inpatient Discharge Record				
	Frequency	Percent	Percent Valid	Cumulative Percent
5503	1	0.4	0.4	94.5
8774	5	1.8	1.8	98.9
Procedure 3 From Inpatient Discharge Record				
	Frequency	Percent	Percent Valid	Cumulative Percent
5631	1	0.4	0.4	97.1
8774	5	1.8	1.8	98.9
8775	1	0.4	0.4	99.3
9851	1	0.4	0.4	100.0
Procedure 4 From Inpatient Discharge Record				
	Frequency	Percent	Percent Valid	Cumulative Percent
5732	1	0.4	0.4	99.3
8774	2	0.7	0.7	100.0
2014				
Principal Procedure From Inpatient Discharge Record				
	Frequency	Percent	Percent Valid	Cumulative Percent
5504	1	0.3	0.3	94.8
8774	1	0.3	0.3	96.9
9851	1	0.3	0.3	99.1
Procedure 2 From Inpatient Discharge Record				
	Frequency	Percent	Percent Valid	Cumulative Percent
5503	1	0.3	0.3	95.1
5631	3	0.9	0.9	96.0
8774	2	0.6	0.6	98.5
Procedure 3 From Inpatient Discharge Record				
	Frequency	Percent	Percent Valid	Cumulative Percent
5732	1	0.3	0.3	97.6
Procedure 4 From Inpatient Discharge Record				
	Frequency	Percent	Percent Valid	Cumulative Percent
8774	1	0.3	0.3	99.7

Total charges for ED and inpatient services of nephrolithiasis patients with IBS were assessed. The mean for total charges is $27,997 in 2012, $25,207 in 2013, and $27,054 in 2014. The significance of cost for IBS patients admitted to the hospital for nephrolithiasis represents additional care as well as diagnostic and therapeutic procedures.

Multiple regression analysis

A standard multiple regression was performed on the HCUP NEDS 2012, 2013, and 2014 datasets for nephrolithiasis patients with IBS. This method allows for all independent, or predictor, variables to be simultaneously entered into the model. Independent variables are each evaluated in terms of their predictive power in comparison to that of the other independent variables. Insight is provided where each of the independent variables may explain unique variances in a dependent variable.

In the 2012 HCUP NEDS dataset linear regression model tested, the primary diagnosis of nephrolithiasis was utilized as the dependent variable, while age, gender, comorbidities, principal and secondary procedures from the ED, as well as principal and secondary procedures from the inpatient discharge record were utilized as independent variables. The P-value for the principal procedure from the inpatient discharge record rejects the null hypothesis that these procedures happen by chance, with a return of 0.001 (see Table 9). This information demonstrates that the principal procedure from the inpatient discharge record represents a statistically significant contribution. Principal procedures include medical imaging modalities such as ultrasound and CT as well as minor and/or major procedures and interventions. Based on the information provided in the descriptive statistical analysis of IBS patients in 2012, ureteroscopy and cystoscopy were the only principal procedures returned related to nephrolithiasis demonstrating the significance of these procedures to nephrolithiasis in IBS patients. While the 2013 dataset does not provide the same results, the 2014 dataset rejects the null hypothesis with a P-value of 0.024 for the principal procedure from the ED (see Table 10). The information provided in the descriptive statistical analysis of IBS patients in 2014 returned CT of the abdomen as the most common procedure in the ED. These results from the linear regression models in 2012 and 2014 require additional regression models of analysis to better understand the data and determine which variables may have higher predictability.

**Table 9:** Linear Regression of IBS for 2012 IBS, irritable bowel syndrome; Std, standard; Sig, significance. ^a^Dependent Variable: Diagnosis 1.

Coefficients^a^								
		Unstandardized Coefficients		Standardized Coefficients			95.0% Confidence Interval for B	
Model		B	Std. Error	Beta	t	Sig	Lower Bound	Upper Bound
1	(Constant)	32.604	3.006		10.846	.000	26.692	38.517
	Age in years at admission	-0.009	0.040	-0.012	-0.222	0.825	-0.086	0.069
	Indicator of Sex	1.381	1.298	0.056	1.063	0.288	-1.173	3.934
	Principal procedure from ED	0.522	0.368	0.091	1.421	0.156	-0.201	1.246
	Procedure 2 from ED	-0.961	0.809	-0.076	-1.189	0.235	-2.551	0.629
	Principal procedure from inpatient discharge record	-0.902	0.278	-0.241	-3.241	0.001	-1.450	-0.355
	Procedure 2 from inpatient discharge record	0.349	0.298	0.087	1.173	0.242	-0.236	0.935
	Diagnosis 2	0.026	0.038	0.037	0.685	0.494	-0.048	0.100
	Diagnosis 3	0.003	0.031	0.005	0.084	0.933	-0.058	0.063
	Diagnosis 4	-1.819E-5	0.030	0.000	-0.001	1.000	-0.058	0.058

**Table 10:** Linear Regression of IBS for 2014 IBS, irritable bowel syndrome; Std, standard; Sig, significance. ^a^Dependent Variable: Diagnosis 1.

Coefficients^a^								
		Unstandardized Coefficients		Standardized Coefficients			95.0% Confidence Interval for B	
Model		B	Std. Error	Beta	t	Sig	Lower Bound	Upper Bound
1	(Constant)	19.247	2.130		9.037	0.000	15.057	23.437
	Age in years at admission	0.026	0.029	0.051	0.912	0.363	-0.030	0.083
	Indicator of Sex	1.578	0.873	0.101	1.808	0.072	-0.140	3.295
	Diagnosis 2	0.048	0.031	0.087	1.567	0.118	-0.012	0.109
	Diagnosis 3	0.025	0.018	0.077	1.356	0.176	-0.011	0.061
	Diagnosis 4	-0.011	0.019	-0.034	-0.596	0.551	-0.049	0.026
	Principal procedure from ED	-1.326	0.584	-0.170	-2.270	0.024	-2.474	-0.177
	Procedure 2 from ED	0.611	0.606	0.075	1.008	0.314	-0.581	1.803
	Principal procedure from inpatient discharge record	-0.091	0.175	-0.041	-0.520	0.603	-0.435	0.253
	Procedure 2 from inpatient discharge record	-0.248	0.277	-0.070	-0.897	0.371	-0.794	0.297

A stepwise regression model was then tested with age as the dependent variable as it has been determined to be a contributing factor of nephrolithiasis and gender, diagnoses, principal procedures from the ED, and principal procedures from the inpatient discharge record as independent variables. This stepwise regression allows SPSS to automatically select variables based on statistical criteria and enter them in the order of the equation. Although this method can be controversial for its false positive errors, in machine learning, stepwise procedures have been utilized with remarkable success. 

The 2012 HCUP NEDS dataset yields an adjusted R square of 0.011, demonstrating that 1% of the variance of age (dependent variable) is explained by independent variables. These results determine that age does not influence the principal procedures performed, including ultrasound vs. CT; however, it does not indicate that age does not contribute to nephrolithiasis. The normal probability plot (P-P) for age does not suggest any major deviations from normality demonstrating age as a standardized result. In the 2013 and 2014 HCUP NEDS datasets, age is normalized against the 2012 dataset results with R square (0.061 or 6% in 2013 and 0.026 or 2% in 2014) and P-Plots showing very similar results.

The principal procedure class was then assessed using the stepwise model of regression. This method allowed for the assessment of independent variables such as primary diagnosis and comorbidity to predict outcomes. In the 2012 analysis, procedures play a role in predictability which is seen in values of 0.002 and 0.035 (see Table 11), and in 2013 although not statistically significant, the P-value for the principal procedure from the inpatient discharge record returned 0.054 (see Table 11). In 2014, comorbidity played a role in predictability which can be seen in the P-value as 0.025 (see Table 11). While there is variability in these stepwise regression models, the data provided give insight into the diagnostic and therapeutic procedures for IBS patients, a key component of this study.

**Table 11:** Stepwise Regression of Principal Procedure Class for Inpatients With IBS Std, standard; sig, significance; IBS, irritable bowel syndrome. ^a^Dependent variable: Principal procedure class for inpatient procedure.

Coefficients^a^								
2012								
		Unstandardized Coefficients		Standardized Coefficients			95.0% Confidence Interval for B	
Model		B	Std. Error	Beta	t	Sig	Lower Bound	Upper Bound
1	(Constant)	0.703	0.763		0.921	0.364	-0.853	2.259
	Diagnosis 1	0.024	0.015	0.212	1.629	0.113	-0.006	0.054
	Diagnosis 2	0.001	0.009	0.009	0.080	0.937	-0.017	0.019
	Diagnosis 3	-0.004	0.007	-0.065	-0.544	0.590	-0.019	0.011
	Diagnosis 4	-0.007	0.007	-0.122	-1.022	0.315	-0.020	0.007
	Principal procedure from inpatient discharge record	-0.020	0.028	-0.093	-0.719	0.478	-0.077	0.037
	Procedure 2 from inpatient discharge record	-0.023	0.028	-0.137	-0.792	0.434	-0.081	0.036
	Procedure 3 from inpatient discharge record	0.225	0.064	0.576	3.481	0.002	0.093	0.356
	Procedure 4 from inpatient discharge record	0.266	0.121	0.334	2.205	0.035	0.020	0.513
2013								
		Unstandardized Coefficients		Standardized Coefficients			95.0% Confidence Interval for B	
Model		B	Std. Error	Beta	t	Sig	Lower Bound	Upper Bound
1	(Constant)	0.911	1.305		0.698	0.492	-1.796	3.618
	Diagnosis 1	0.013	0.024	0.085	0.532	0.600	-0.037	0.062
	Diagnosis 2	-0.008	0.012	-0.108	-0.667	0.512	-0.034	0.017
	Diagnosis 3	0.009	0.019	0.084	0.466	0.646	-0.031	0.049
	Diagnosis 4	0.005	0.018	0.047	0.255	0.801	-0.033	0.042
	Principal procedure from inpatient discharge record	0.143	0.070	0.388	2.037	0.054	-0.003	0.289
	Procedure 2 from inpatient discharge record	0.084	0.084	0.192	0.995	0.330	-0.091	0.258
	Procedure 3 from inpatient discharge record	0.210	0.139	0.290	1.515	0.144	-0.078	0.498
	Procedure 4 from inpatient discharge record	-0.659	0.453	-0.271	-1.456	0.160	-1.597	0.280
2014								
		Unstandardized Coefficients		Standardized Coefficients			95.0% Confidence Interval for B	
Model		B	Std. Error	Beta	t	Sig	Lower Bound	Upper Bound
1	(Constant)	1.872	1.222		1.532	0.139	-0.650	4.394
	Diagnosis 1	-0.005	0.028	-0.034	-0.195	0.847	-0.063	0.052
	Diagnosis 2	-0.021	0.019	-0.185	-1.070	0.295	-0.060	0.019
	Diagnosis 3	0.028	0.012	0.412	2.392	0.025	0.004	0.052
	Diagnosis 4	-0.012	0.010	-0.204	-1.188	0.246	-0.033	0.009
	Principal procedure from inpatient discharge record	0.053	0.045	0.241	1.186	0.247	-0.040	0.146
	Procedure 2 from inpatient discharge record	0.028	0.049	0.113	0.566	0.577	-0.074	0.130
	Procedure 3 from inpatient discharge record	0.383	0.207	0.415	1.855	0.076	-0.043	0.810
	Procedure 4 from inpatient discharge record	-1.253	0.635	-0.398	-1.973	0.060	-2.563	0.057

Additional stepwise regression models were analyzed with total charges as the dependent variable, while age, gender, and procedure class were independent variables. The 2012 stepwise regression model for total charges for ED services yields a P-value of 0.000 for the principal procedure class for major therapeutic procedures which include interventions requiring the use of the operating room (OR), and a P-value of 0.017 for minor diagnostic procedures which include ultrasound and CT imaging (see Table 12). These P-values reject the null hypothesis that major therapeutic and minor diagnostic procedures and interventions happen by chance and represent statistically significant contributions to total charges for ED visits. The adjusted R-square goes up slightly with the addition of minor diagnostic procedures by 0.092.

**Table 12:** Stepwise Regression of Total Charges for ED Services 2012 Std, standard; sig, significance; ED, emergency department. ^a^Dependent variable: Total charge for ED services.

Coefficients^a^								
		Unstandardized Coefficients		Standardized Coefficients			95.0% Confidence Interval for B	
Model		B	Std. Error	Beta	t	Sig	Lower Bound	Upper Bound
1	(Constant)	5208.518	268.249		19.417	0.000	4680.630	5736.405
	PCLASS_MAJORTX_ED	22980.482	4661.665	0.274	4.930	0.000	13806.779	32154.186
2	(Constant)	5078.792	271.632		18.697	0.000	4544.239	5613.345
	PCLASS_MAJORTX_ED	23110.208	4625.730	0.275	4.996	0.000	14007.097	32213.320
	PCLASS_MinorTX_ED	3253.958	1360.423	0.132	2.392	0.017	576.742	5931.175

The 2012 stepwise model with total charges for ED and inpatient services as the dependent variable yields P-values of 0.006 and 0.001 for minor therapeutic non-OR procedures, as well as a P-value of 0.018 for minor diagnostic procedures, which again include ultrasound and CT imaging (see Table 13). These P-values reject the null hypothesis that these minor therapeutic and minor diagnostic procedures happen by chance and represent statistically significant contributions to total charges for ED and inpatient services. The adjusted R-square goes up slightly with the addition of minor diagnostic procedures by 0.147. 

**Table 13:** Stepwise Regression of Total Charges for ED and Inpatient Services 2012 ED, emergency department; std, standard; sig, significance. ^a^Dependent variable: Total charge for ED and inpatient services.

Coefficients^a^								
		Unstandardized Coefficients		Standardized Coefficients			95.0% Confidence Interval for B	
Model		B	Std. Error	Beta	t	Sig	Lower Bound	Upper Bound
1	(Constant)	24591.547	3427.299		7.175	0.000	17757.706	31425.388
	PCLASS_MinorTX_IP	27619.342	9760.953	0.318	2.830	0.006	8156.555	47082.129
2	(Constant)	18176.359	4248.106		4.279	0.000	9703.779	26648.939
	PCLASS_MinorTX_IP	34034.530	9810.580	0.392	3.469	0.001	14467.946	53601.114
	PCLASS_MinorDX_IP	16422.881	6796.969	0.273	2.416	0.018	2866.754	29979.008

The 2013 stepwise regression model for total charges for ED services yields a P-value of 0.000 for the principal procedure class for major therapeutic procedures which include interventions requiring the use of the OR, and a P-value of 0.006 for minor therapeutic, or non-OR, procedures (see Table 14). These P-values reject the null hypothesis that major therapeutic and minor therapeutic procedures and interventions happen by chance and represent statistically significant contributions to total charges for ED visits. The adjusted R-square goes up slightly with the addition of minor therapeutic procedures by 0.156.

**Table 14:** Stepwise Regression of Total Charges for ED Services 2013 std, standard; sig, significance; ED, emergency department. ^a^Dependent variable: Total charge for ED.

Coefficients^a^								
		Unstandardized Coefficients		Standardized Coefficients			95.0% Confidence Interval for B	
Model		B	Std. Error	Beta	t	Sig	Lower Bound	Upper Bound
1	(Constant)	4995.332	281.106		17.770	0.000	4441.341	5549.323
	PCLASS_MAJORTX_ED	24577.668	4197.804	0.366	5.855	0.000	16304.820	32850.516
2	(Constant)	4857.417	281.464		17.258	0.000	4302.706	5412.129
	PCLASS_MAJORTX_ED	24715.583	4136.664	0.369	5.975	0.000	16563.023	32868.143
	PCLASS_MinorTX_ED	4373.869	1585.079	0.170	2.759	0.006	1249.987	7497.750

The 2013 stepwise model with total charges for ED and inpatient services as the dependent variable yields P-values of 0.004 for gender as an independent variable and rejects the null hypothesis and represents a statistically significant contribution to total charges for ED and inpatient services (see Table 15).

**Table 15:** Stepwise Regression of Total Charges for ED and Inpatient Services 2013 std, standard; sig, significance; ED, emergency department. ^a^Dependent Variable: Total charge for ED and Inpatient services

Coefficients^a^								
		Unstandardized Coefficients		Standardized Coefficients			95.0% Confidence Interval for B	
Model		B	Std. Error	Beta	t	Sig	Lower Bound	Upper Bound
1	(Constant)	34462.750	3487.699		9.881	0.000	27504.981	41420.519
	Indicator of sex	-11948.914	3962.660	-0.341	-3.015	0.004	-19854.204	-4043.624

The 2014 stepwise regression model for total charges for ED services yields a P-value of 0.000 for the principal procedure class for major therapeutic procedures which include interventions requiring the use of the OR (see Table 16). This P-value rejects the null hypothesis and represents a statistically significant contribution to total charges for ED visits. 

**Table 16:** Stepwise Regression of Total Charges for ED Services 2014 std, standard; sig, significance; ED, emergency department. ^a^Dependent variable: Total charge for ED.

Coefficients^a^								
		Unstandardized Coefficients		Standardized Coefficients			95.0% Confidence Interval for B	
Model		B	Std. Error	Beta	t	Sig	Lower Bound	Upper Bound
1	(Constant)	6371.311	329.259		19.350	0.000	5723.154	7019.468
	PCLASS_MAJORTX_ED	23037.689	5509.553	0.243	4.181	0.000	12191.948	33883.431

The 2014 stepwise model with total charges for ED and inpatient services as the dependent variable yields P-values of 0.001 for major therapeutic, OR procedures. This P-value rejects the null hypothesis and represents a statistically significant contribution to total charges for ED and inpatient services.

The 2012, 2013, and 2014 stepwise regression analyses with total charges as the dependent variable and gender and procedure class as independent variables contributing to the model confirm the hypothesis that patients with a comorbidity of IBS who are assessed with ultrasound before CT have poorer outcomes and accrue greater healthcare costs, due to additional imaging and the need for intervention.

## Discussion

This study analyzed the HCUP NEDS 2012, 2013, and 2014 database variables including age, gender, and comorbidities of obesity, diabetes, and IBS, as well as dehydration and hyperosmolality, relevant medical imaging exams, interventions and procedures, total charges, and discharge versus admission. The study sample included 164,410 ED visits for nephrolithiasis in 2012, 163,254 in 2013, and 181,528 in 2014 for a total of 509,192 visits.

The prevalence of nephrolithiasis is highest among adults between the ages of 30 and 42 and was found to be more common in males than females. While there is a slight prevalence of nephrolithiasis in patients with a comorbidity of obesity (0.57%), there is a higher rate in those with diabetes (4.78%). However, the focus of this study is on patients with IBS, as this population is at greater risk of dehydration due to regular bouts of diarrhea. 

The link between nephrolithiasis and dehydration has been clearly outlined. While this link seems to be well known, there is a lack of studies on laboratory values for dehydration, such as plasma osmolality, which is a strong indicator of chronic dehydration. While dehydration is found in patients across the databases utilized in this study, there is a lack of confidence that this is consistently assessed, if at all, upon presentation to the ED and therefore most likely does not accurately represent the prevalence in the IBS population studied, and more importantly stone formers.

Overall data represent ED visits for nephrolithiasis between 2012 and 2014, which often includes medical imaging for assessment and diagnosis. The prevalence of ultrasound and CT exams was reviewed for both ED cases and those admitted to the hospital. Where ultrasound and CT are seen in the same visit, it can be assumed that ultrasound was performed first as this method of assessing patients is more frequently utilized despite the ACR's appropriateness criteria guidelines. Non-contrast CT is the first-line imaging choice for detecting nephrolithiasis with a 95% sensitivity and specificity and is considered the gold standard for its ability to identify the location, size, and composition of stones, and its ability to provide differential diagnoses. 

Ultrasound of the urinary system was more commonly utilized in the ED than CT; however, CT was more commonly utilized for patients admitted to the hospital with nephrolithiasis. This is interesting as those that are admitted to the hospital due to nephrolithiasis are due to larger stone size, requiring intervention, blood, or infection. 

Analysis of procedure class for inpatient procedures yielded 9158 minor and major diagnostic and therapeutic procedures in 2012, 8542 in 2013, and 9628 in 2014. Total charges for patients with nephrolithiasis presenting to the ED and then being admitted to the hospital were found to be significantly higher than that of patients discharged from the ED. In 2012, the mean total charges for ED services were $21,796 less than the combined ED and inpatient charges; in 2013, the mean total charges for ED services were $23,557 less than the combined ED and inpatient charges; and in 2014, the mean total charges for ED services were $26,555 less than the combined ED and inpatient charges. This is a significant burden of cost to patients and healthcare systems. 

IBS was assessed as a comorbidity of nephrolithiasis in this study as dehydration is very common in patients with recurrent bouts of diarrhea. Three hundred and fifty-nine patients were identified in 2012 with nephrolithiasis and IBS, 272 patients in 2013, and 327 patients in 2014 with a predominance of females (70% female versus 30% male) within this population. Somewhat shocking to this study was that within this patient population from 2012 to 2014, zero patients had ultrasound imaging and only 22 patients had a CT. While medical imaging is low in this group, 104 (10.9%) required an intervention and/or procedure, and of the 104, 44 interventions (42%) were directly related to stone removal. The mean total charge for nephrolithiasis patients with IBS admitted to the hospital from the ED is $26,753, which is like that of the non-IBS nephrolithiasis population. 

Clinical decision-making is an integral part of patient outcomes and its accuracy is a necessary entity in today’s healthcare environment. Several variables in this study were compared to better understand the prevalence of nephrolithiasis and to analyze and investigate a predictive model for improving patient outcomes of nephrolithiasis. Statistical analyses of the HCUP NEDS 2012, 2013, and 2014 datasets were done using descriptive statistics for frequencies and percentages, as well as multiple regression analysis including linear and stepwise regression models. 

While descriptive statistics of this study align with the literature review of medical imaging in nephrolithiasis patients, ultrasound being utilized more frequently than CT upon presentation to the ED, which confirms the lack of adherence to the ACR’s guidelines, and the lack of laboratory testing and/or results available in the dataset prove difficult to validate adherence to the recommended laboratory studies and metabolic evaluations. Multiple regression models demonstrate that principal procedures, which include medical imaging modalities such as ultrasound and CT, as well as minor and or major procedures and interventions, from the inpatient discharge record represent a statistically significant contribution to nephrolithiasis. Based on the information provided in the descriptive statistical analysis of IBS, a variety of principal procedures returned were related to nephrolithiasis demonstrating the significance of these to nephrolithiasis in IBS patients. 

This study shows that although many patients are discharged directly from the ED, there is a significant number of patients admitted to the hospital for nephrolithiasis, many of whom were assessed with ultrasound first. This confirms the hypothesis that patients assessed with ultrasound before CT have poorer outcomes and accrue greater healthcare costs. 

Certain comorbidities associated with nephrolithiasis may produce larger stones due to chronic dehydration; however, due to the lack of available data within the HCUP NEDS datasets on plasma osmolality, this theory is difficult to confirm. We can see that obesity, diabetes, and IBS are all contributing factors to nephrolithiasis risk and prove the hypothesis that certain comorbidities are most closely linked to nephrolithiasis. Interventions in IBS patients are often more invasive and fall into the minor diagnostic and therapeutic procedure class categories, as well as a large number in the major therapeutic category. Patients with IBS have a two-fold likelihood of requiring an intervention than that of the general nephrolithiasis population, resulting in poorer outcomes and greater healthcare costs.

Hydration is a significant factor for kidney stone formers, and the literature highlights the need for increased fluids in prevention. However, there is little information available in the HCUP NEDS datasets for dehydration assessment upon presentation to the ED. Prevention of nephrolithiasis would release a significant healthcare burden on the patient and the national healthcare system. Increased fluid intake should increase urine output and ensure mechanical diuresis to prevent the stagnation of organic materials and the formation of stones. Increased water intake has resulted in the attenuation of circulating copeptin, serving as a simple and cost-effective intervention to reduce circulating vasopressin, which conserves water and decreases urine volume [8]. Chronic dehydration can be assessed with plasma osmolality and copeptin levels and should be considered as part of the initial assessment upon presentation to the ED with suspected nephrolithiasis.

Limitations of study

The HCUP NEDS database provided insight and results in this study of nephrolithiasis; however, it is limited due to its retrospective nature. The databases are missing information about ED charges for a variety of weighted percentages, 15% for 2012 and 2013, and 16% for 2014 [5-7], and focuses on entry-level versus patient-level records. Therefore, patients who may revisit the ED multiple times each year may be present as separate entries. The NEDS is also limited by direct admission from the ED to the hospital; only one discharge record is included making differentiation of procedures performed in the ED versus part of the inpatient stay unattainable. 

There is potential misclassification of variables inherent to administrative data which relies heavily on diagnosis codes entered by individual hospital personnel. Because IBS is not always reported, or diagnosed, the overall data may be incomplete or exposed to bias. Therefore, this exploratory study and analysis may be skewed. Additionally, the lack of ICD-9 coding for plasma osmolality, and perhaps the sheer lack of testing, prevent this study from confirming the hypothesis of plasma osmolality and copeptin levels as a link to nephrolithiasis risk and stone formation.

Despite these limitations, the HCUP NEDS datasets allow for a national perspective of trending in nephrolithiasis patient populations and have provided a great deal of insight.

## Conclusions

The HCUP NEDS datasets for 2012, 2013, and 2014 have provided insight into the prevalence of nephrolithiasis based on age, gender, comorbidity, diagnostic assessment, and procedures or interventions required during ED and hospital care. The highest rate of nephrolithiasis occurrence is between 30 and 42 years of age and is more common in the general population of males. However, female patients among the same age group with IBS have a higher prevalence of nephrolithiasis than males. Adherence to medical imaging guidelines and laboratory testing for nephrolithiasis is lacking. This study demonstrates that patients assessed with ultrasound first are more likely to be admitted to the hospital than those assessed following the ACR’s recommended low-dose CT imaging and accrue greater healthcare costs overall. Although many studies outline this lack of adherence to the ACR’s appropriateness criteria, there are no available reports on patient outcomes or hospital admissions. This research is the first to report on patient outcomes, specifically IBS as a comorbidity, as well as the prevalence of procedures and interventions in nephrolithiasis, and total charges for ED versus inpatient hospital care. 
